# 33. Evaluation of Rural-Urban Differences in Hospitalization and Mortality Rates for US COVID-19 Patients in the United States

**DOI:** 10.1093/ofid/ofab466.033

**Published:** 2021-12-04

**Authors:** Alfred (Jerrod) Anzalone, Ronald Horswell, San Chu, Brian Hendricks, Jeremy Harper, William Beasley, Lucio Miele, Clifford james Rosen, James McClay, Sally L Hodder

**Affiliations:** 1 University of Nebraska Medical Center, Omaha, NE; 2 Pennington Biomedical Research Centre, Baton Rouge, Louisiana; 3 Pennington Biomedical Research Center, Baton Rouge, Louisiana; 4 West Virginia University, Morgantown, West Virginia; 5 Owl Health Works, Indianapolis, Indiana; 6 University of Oklahoma, Norman, Oklahoma; 7 LSUHSC School of Medicine, New Orleans, New Orleans, Louisiana; 8 Maine Medical Center Research Institute, scarborough, Maine; 9 West Virginia University School of Medicine, Morgantown, West Virginia

## Abstract

**Background:**

Rural communities are among the most vulnerable and resource-scarce populations in the United States. Rural data is rarely centralized, precluding comparability across regions, and no significant studies have studied this population at scale. The purpose of this study is to present findings from the National COVID Cohort Collaborative (N3C) to provide insight into future research and highlight the urgent need to address health disparities in rural populations.

N3C Patient Distribution

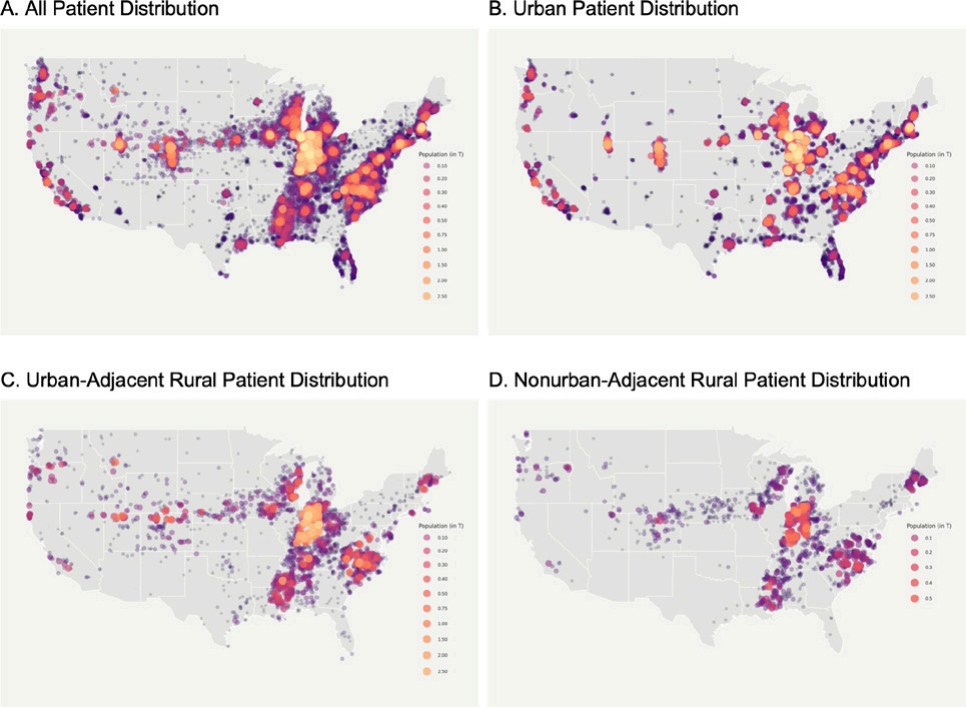

This figure shows the geospatial distribution of the N3C COVID-19 positive population. N3C contains data from 55 data contributors from across the United States, 40 of whom include sufficient location information to map by ZIP Code centroid spatially. Of those sites, we selected 27 whose data met our minimum robustness qualifications for inclusion in our study. This bubble map is to scale with larger bubbles representing more patients. A. shows all N3C patients. B. shows only urban N3C distribution. C. shows the urban-adjacent rural patient distribution. D. shows the nonurban-adjacent rural patient distribution, representing the most isolated patients in N3C.

**Methods:**

This retrospective cohort of 573,018 patients from 27 hospital systems presenting with COVID-19 between January 2020 and March 2021, of whom 117,897 were admitted (see Data Analysis Plan diagram for inclusion/exclusion criteria), analyzes outcomes and 30-day survival for the hospitalized population by the degree of rurality.

Multivariate Cox regression analysis and mixed-effects models were used to estimate the association between rurality, hospitalization, and all-cause mortality, controlling for major risk factors associated with rural-urban health discrepancies and differences in health system outcomes. The difference in distribution by rurality is described as well as supplemented by population-level statistics to confirm representativeness.

Data Analysis Plan

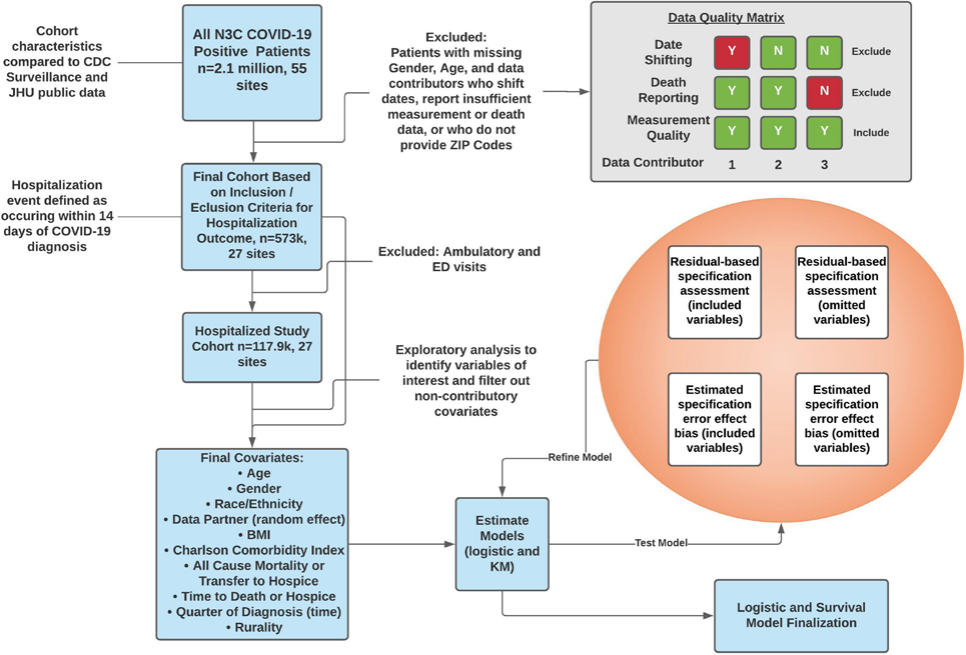

This data analysis plan includes an overview of study inclusion and exclusion criteria, the matrix for data robustness to determine potential sites to include, and our covariate selection, model building, and residual testing strategy.

**Results:**

This study demonstrates a significant difference between hospital admissions and outcomes in urban versus urban-adjacent rural (UAR) and nonurban-adjacent rural (NAR) lines. Hospital admissions for UAR (OR 1.41, p< 0.001, 95% CI: 1.37 – 1.45) and NAR (OR 1.42, p< 0.001, 95% CI: 1.35 – 1.50) were significantly higher than their urban counterparts. Similar distributions were present for all-cause mortality for UAR (OR 1.39, p< 0.001, 95% CI: 1.30 – 1.49) and NAR (OR 1.38, p< 0.001, 95% CI: 1.22 – 1.55) compared to urban populations. These associations persisted despite adjustments for significant differences in BMI, Charlson Comorbidity index Score, gender, age, and the quarter of diagnosis for COVID-19.

Baseline Characteristics Hospitalized COVID-19 Positive Population by Rurality Category, January 2020 – March 2021

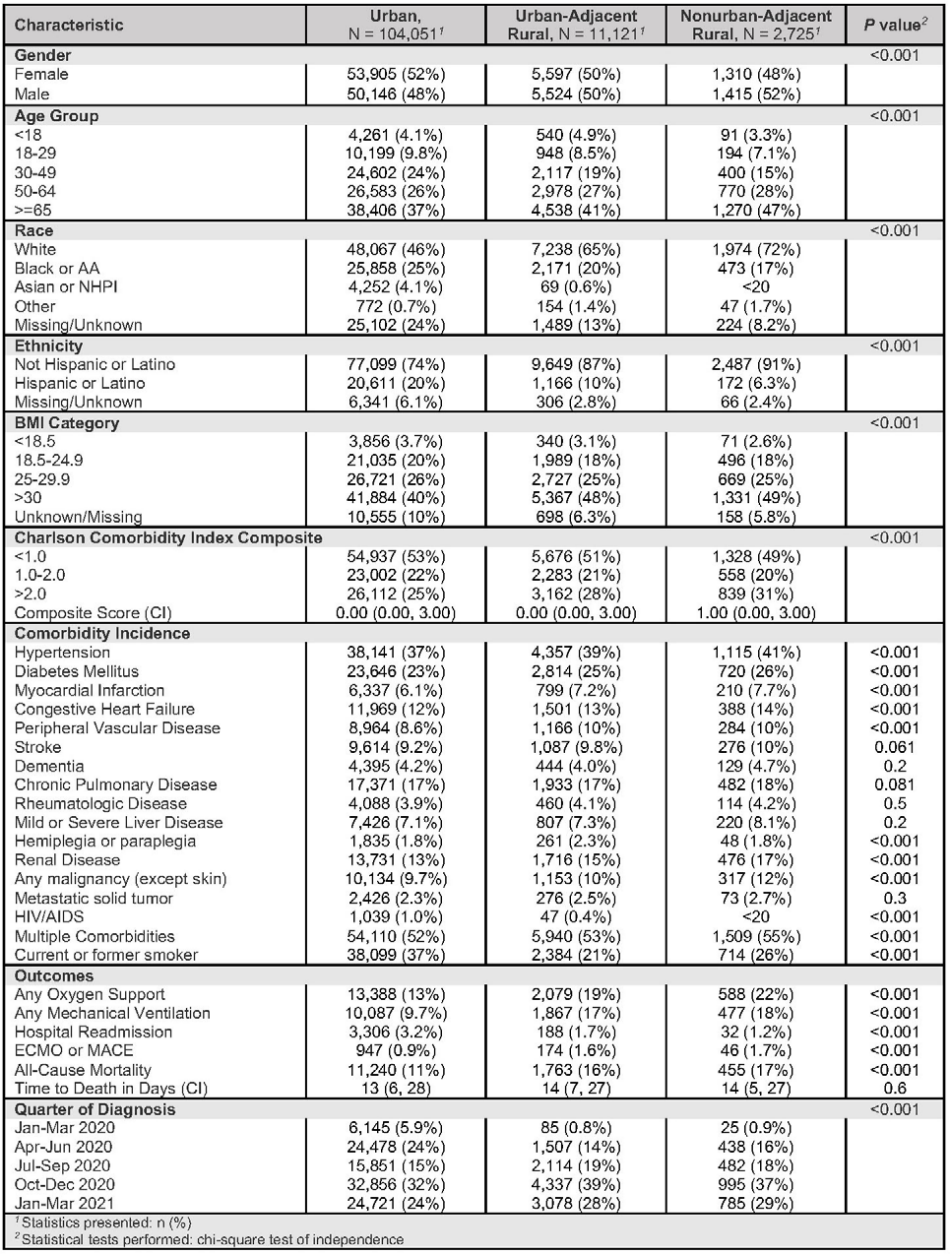

Survival Curves in Hospitalized Patients Over 30 Days from Day of Admission

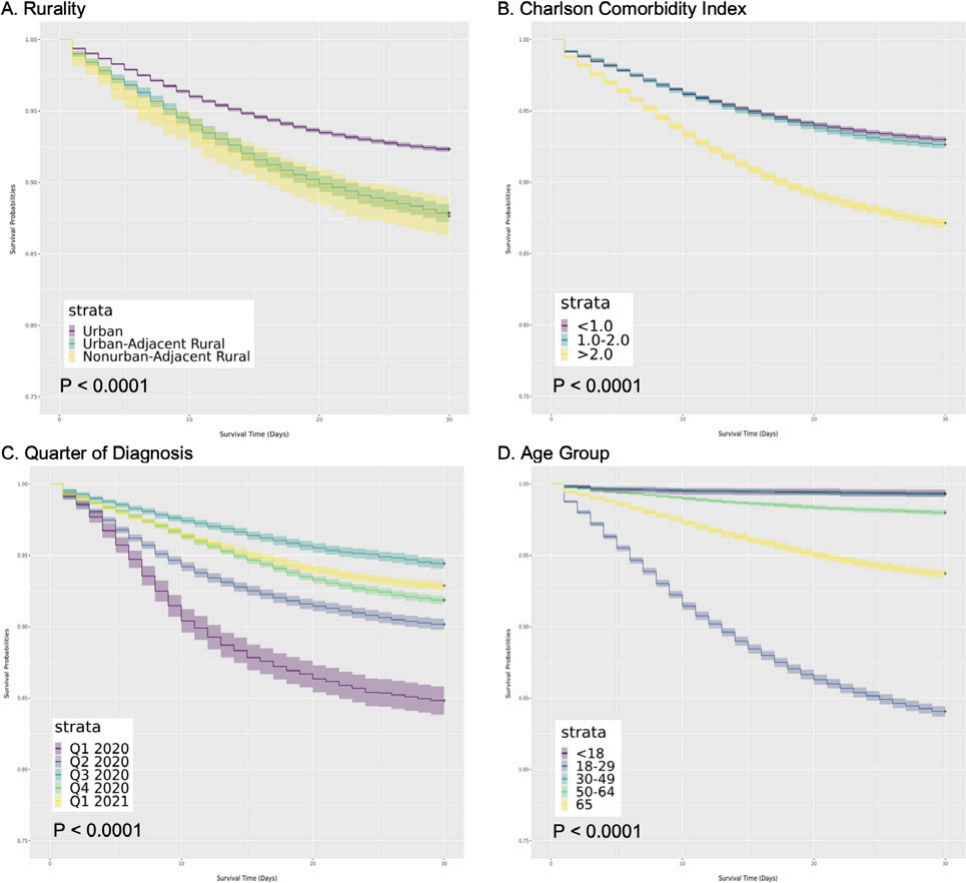

This figure shows a survival plot of COVID-19 positive hospitalized patients in N3C by rural category (A), Charlson Comorbidity Index (B), Quarter of Diagnosis (C), and Age Group (D) from hospital admission through day 30. Events were censored at day 30 based on the incidence of death or transfer to hospice care. These four factors had the highest predictive power of the covariates evaluated in this study.

Unadjusted and Adjusted Odds Ratios for Hospitalization and All-Cause Mortality by Rural Category, January 2020 – March 2021

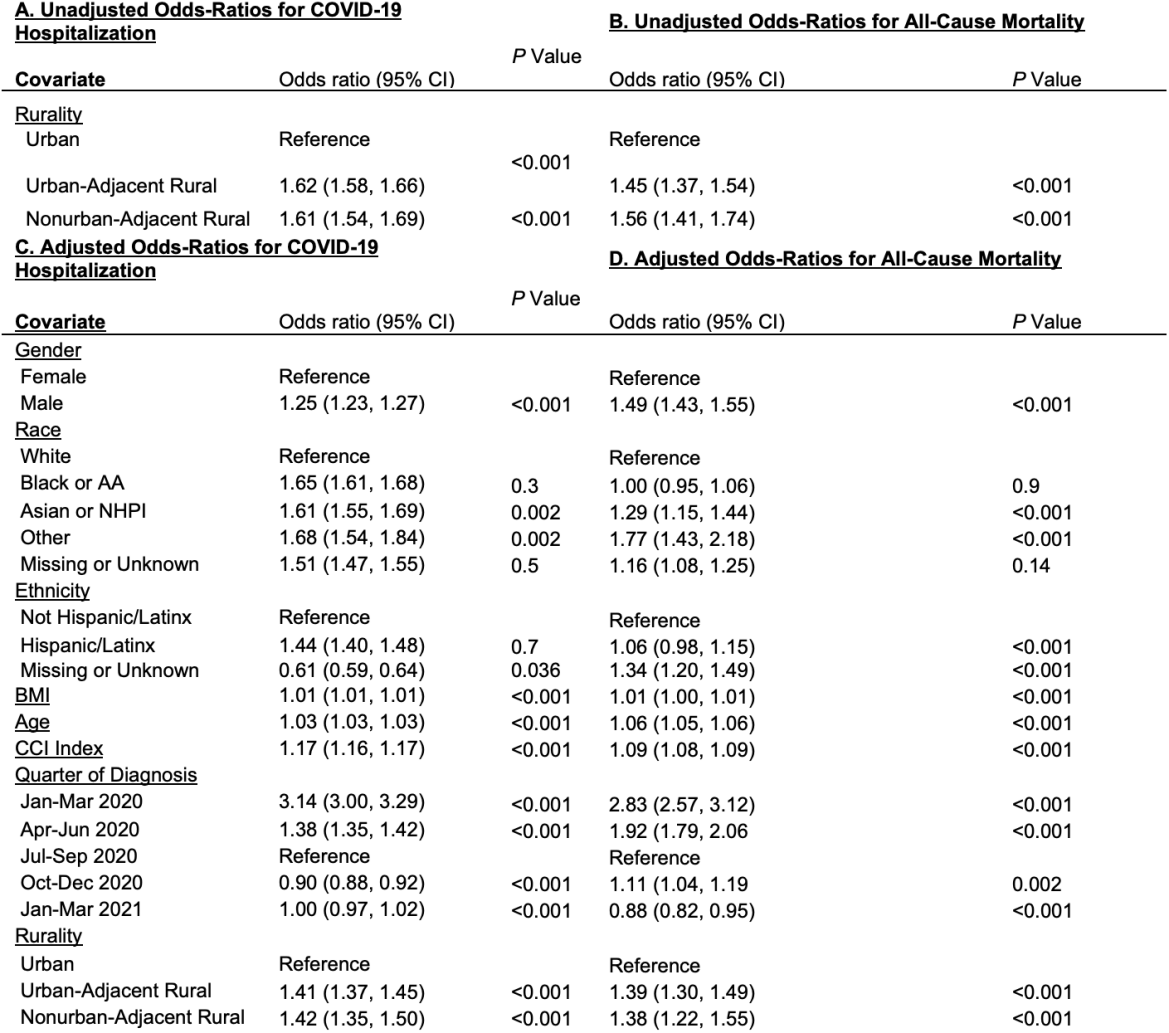

This figure shows the adjusted and unadjusted odds ratios for being hospitalized or dying after hospitalization for the COVID-19 positive population in N3C. Risk is similar between adjusted and unadjusted models, suggesting a real impact of rurality on all-cause mortality. A shows the unadjusted odds ratios for admission to the hospital after a positive COVID-19 diagnosis for all N3C patients. B shows the unadjusted odds ratios for all-cause mortality at any point after hospitalization for COVID-19 positive patients. C shows the adjusted odds ratios for being admitted to the hospital after a positive COVID-19 diagnosis for all N3C patients. D shows the adjusted odds ratios for all-cause mortality for all-cause mortality at any point after hospitalization for COVID-19 positive patients. Adjusted models include adjustments for gender, race, ethnicity, BMI, age, Charlson Comorbidity Index (CCI) composite score, rurality, and quarter of diagnosis. The data provider is included as a random effect in all models.

**Conclusion:**

In N3C, we found that hospitalizations and all-cause mortality were greater among rural populations when compared to urban populations after adjustment for several factors, including age and co-morbidities. This study also identified key demographic and clinical disparities among rural patients that require further investigation.

**Disclosures:**

**Sally L. Hodder, M.D.**, **Gilead** (Advisor or Review Panel member)**Merck** (Grant/Research Support, Advisor or Review Panel member)**Viiv Healthcare** (Grant/Research Support, Advisor or Review Panel member)

